# AUTONOMY, NATALITY AND FREEDOM: A LIBERAL RE-EXAMINATION OF HABERMAS IN THE ENHANCEMENT DEBATE

**DOI:** 10.1111/bioe.12082

**Published:** 2014-02-19

**Authors:** Jonathan Pugh

**Keywords:** enhancement, autonomy, Habermas

## Abstract

Jurgen Habermas has argued that carrying out pre-natal germline enhancements would be inimical to the future child's autonomy. In this article, I suggest that many of the objections that have been made against Habermas' arguments by liberals in the enhancement debate misconstrue his claims. To explain why, I begin by explaining how Habermas' view of personal autonomy confers particular importance to the agent's embodiment and social environment. In view of this, I explain that it is possible to draw two arguments against germline enhancements from Habermas' thought. I call these arguments ‘the argument from negative freedom’ and ‘the argument from natality’. Although I argue that many of the common liberal objections to Habermas are not applicable when his arguments are properly understood, I go on to suggest ways in which supporters of enhancement might appropriately respond to Habermas' arguments.

Germline modification technologies promise to allow parents to determine that their child have the genes for certain mental and physical traits. As well as having obvious benefits for the treatment of inheritable genetic diseases, many champion the use of these technologies as a means of enhancing future generations by ensuring that they will have the genes for capacities and traits which are conducive to greater well-being for both the enhanced individual and society in general.

However, Jürgen Habermas has contended that determining the genotype of an unborn child by carrying out germline modifications would be inimical to the future child's autonomy. Responses to Habermas in the bioethical literature have been, in the main, dismissive, concise and even hostile. For instance, Buchanan claims that Habermas arguments are not backed up by ‘sound reasoning’,^1^ whilst Harris claims that they are a product of a ‘… bamboozled mind’.^2^ Conversely, supporters of Habermas have argued that liberal bioethicists have misunderstood his arguments, and that they therefore ‘… have some work to do to reply to him’.^3^

I endorse Prusak's claim that liberal bioethicists have thus far failed to provide a decisive objection to Habermas' arguments against germline enhancements; my aim in this article is to suggest areas in which supporters of enhancement might yet raise cogent, although not necessarily decisive, objections to these arguments. In Section I, I shall begin by providing a sketch of Habermas' view of autonomy. In Section II I shall delineate two arguments that Habermas seems to espouse in support of the claim that germline enhancements would pose a threat to the autonomy of enhanced individuals, and I shall explain why some dismissals of these arguments have been premature. Finally, in section III, I shall explain how liberals in the enhancement debate might more adequately respond to Habermas' arguments.

## I. Habermas On Autonomy

Habermas has offered a rich account of autonomy over the course of his career, the nuances of which I cannot fully explore here. Here, I shall draw attention to four central elements undergirding Habermas' understanding of the concept in *The Future of Human Nature*, which are also central to his objections to germline enhancement.

The first element to highlight is the importance that Habermas confers to the agent's embodiment. For Habermas, each person's sense of their own identity is indelibly linked to their existence as an *embodied* being; humans do not merely have a body as a contingent point of fact, but rather exist ‘… as a lived body that enables and empowers (them) to perform actions’.^4^ Accordingly, Habermas claims that our bodies represent the ‘primary mode of our subjective existence’.^5^ What Habermas is claiming here is that individuals subjectively experience their own existence as an agent who can act in the world by virtue of being embodied.

However, Habermas is also aware that our bodies partly determine us, and that this can lead to feelings of self-alienation if the nature of our bodies is in dissonance with our desires. Yet, although it is possible to feel limited by our own bodies, it seems that we can come to assimilate these limitations when we form our plans. Accordingly, we have autonomy, Habermas claims, insofar as we identify with our own bodies, and feel ‘at home’^6^ in them. In doing so, we regard the body not as an externally determining force on our will, but rather as an enabling condition of agency.

Second, it is important to acknowledge that, on Habermas' view, the self is also partly determined by the inter-subjective communication that occurs in one's social environment. He refers to this as the process of ‘socialization’^7^ and claims that the formation of our individual identities is ‘dependent from the moment of birth’^8^ on it. Moreover, Habermas suggests that in forming an identity, we *must* interact with others, since it is through intersubjective communication that we are able to conceive of ourselves as a subject, and also to regard others as subjects worthy of moral consideration. He writes:

Only in the social network of legitimately regulated relations of mutual recognition (can human beings) develop and … maintain a personal identity.^9^

Accordingly, the self's being socially located is essential to the *value* of autonomy on Habermas' view. For Habermas, it is through our regarding each other as members bound into intersubjective relations that we are able to regard each other as free and equal beings in a moral community.

Habermas is thus acutely aware that environmental aspects partially determine the self. We have only a ‘… conditioned freedom, rooted in the organism and the biography of the individual’.^10^ Yet if the notion of an environmentally determined self could tell the *whole* story about ‘who a person is’, then there would seem to be no scope for autonomy. Habermas of course does not endorse this conclusion. In order to evade it, he claims that the ‘conditioned freedom’ of the embodied and socially embedded agent is not inimical to their autonomy, as long as the agent has a conception of their own self that they can identify with in abstraction from these determining influences.

In order to account for how we are to conceive of this abstract sense of self, Habermas incorporates two further significant features into his account of autonomy, by appealing first to Kierkegaarde's concept of ‘being able to be oneself’, and second to Arendt's concept of natality. Habermas claims that in order to conceive of the abstract sense of self mentioned above, we must follow the Kierkegaardian model of the ‘ethically resolute conduct of life’ which demands that:

I *gather* myself and detach myself from the dependencies of an overwhelming environment, jolting myself to the awareness of my individuality and freedom. Once I am emancipated from a self-induced objectification, I also gain distance from myself as an individual.^11^

What Habermas seems to be claiming here is that although the self is partially determined by its environment, there is some element of the self which can ‘detach’ from these dependencies, and which is the source of the agent's individuality and freedom. In failing to acknowledge this aspect of the self, the agent regards herself as wholly determined by her environment, and thus induces an illusion of herself as being merely a passive object, rather than an active subject.

Finally, Habermas also appeals to Arendt's concept of natality in order to explain the possibility of autonomy despite the forces of socialization. Habermas claims that our *natality* (i.e. our being born), confers a uniqueness to each person, representing a point of differentiation between what he terms ‘the socialization fate of a person’ and their ‘natural fate’.^12^ In appealing to the concept of natality, Habermas is able account for the possibility of autonomy despite the forces of socialization, since he claims that our natality allows us to identify the ‘self’ as it existed prior to the forces of socialization. As I shall explain below, this forms the basis for one of his arguments against germline enhancements.

## II. Habermas' Objections to Germline Enhancements

I shall now delineate two arguments that can be abstracted from *The Future of Human Nature* which purport to show that carrying out germline enhancements would threaten the autonomy of enhanced individuals.^13^ The first, I term ‘the empirical argument’, the second I term ‘the argument from natality’.^14^ Finally, I shall explain that Habermas makes a pre-emptive defence against what Malmqvist calls ‘the parents have always done it argument’ in favour of enhancement.^15^

### i) The empirical argument

One argument that is apparent in Habermas' remarks is that enhanced children *might* feel un-free to pursue their own life-projects because of the very fact that they have been enhanced.

*Prima facie*, it might seem odd to claim that enhancement technologies threaten our freedom; in fact, the opposite seems to be true. As Bostrom claims, enhancement technologies might make it the case that the enhanced individual ‘… would enjoy more choice and autonomy in her life, if the modifications were such as to expand her basic capability set’ since they would ‘… open more life-plans than they block’.^16^ As such, the objection that enhancement technologies threaten the child's freedom might seem to be a *non-sequitur*.^17^

However, although enhancements might increase an individual's general freedoms, the problem is that they might also threaten a particular freedom that seems necessary for practical autonomy. Bostrom's argument seems to be that enhancements could increase the enhanced individual's freedom of choice. However, as Dworkin has argued, increasing the number of choices available to an agent may not increase their autonomy; for instance, doing so might change the nature of the agent's choice situation, or involve replacing the agent's preferred option with various other inferior options.^18^ This latter point suggests that a freedom that might be necessary for practical autonomy at the point of action is the freedom to do what one is actually motivated to do.

Habermas seems to argue that germline enhancements could threaten the child's freedom in this sense because of the parental expectations that are implicit in the enhancements. He claims that:

The parent's choice of a genetic program is associated with intentions which later take on the form of expectations addressed to the child.^19^

The crucial point here is that, for Habermas, the parent's choice of enhancement implies their endorsement of a specific life-plan for the child, and an expectation that the child will follow it.

Of course, the parent's choice of enhancement might be in harmony with what the child wants.^20^ Habermas concedes this point; however, he points out that there remains a possibility of ‘dissonant cases’^21^ in which the parental expectation implicit in the enhanced trait is incongruous with the child's desires. Moreover, since the enhanced child would *incarnate* their parent's expectations in embodying an enhanced trait, there is extra weight to his claim that the enhanced child would believe that she could not refuse to act in accordance with these expectations. This is what Habermas seems to have in mind when he claims that in dissonant cases:

Eugenic interventions aiming at enhancement reduce ethical freedom insofar as they tie down the person concerned to rejected but irreversible third party intentions.^22^

### ii) The argument from natality

Habermas also argues that germline interventions would undermine the enhanced child's autonomy because they would undermine the child's ability to conceive of herself as an autonomous agent. A central premise in this argument is Habermas' claim that:

The conditions … of nature-like growth … alone allow us to conceive of ourselves as the authors of our own lives.^23^

As Prusak points out, the idea here is that:

… the contingency of our genetic makeup might well be necessary for a person to be capable of regarding herself as an autonomous being.^24^

To unpack this, recall that for Habermas, the self is a product of its socialization. Nonetheless, Habermas claims that this does not mean that such individuals cannot be autonomous for two reasons. First, he claims that a person's *natality* represents a point of differentiation between a person's ‘socialization fate’ and their ‘natural fate’. Second, he claims that in order for an agent to regard himself as the master of the social forces that presently determine his self-constitution, he must have a conception of himself existing in abstraction from the determination of these forces before he was born, and consequently subjected to these forces. Habermas writes:

For a person to be himself, a point of reference is required which goes back beyond the lines of tradition and the contexts of interaction which constitute the process of formation through which personal identity is moulded in the course of a life history.^25^

This appeal to natality underpins a second objection that Habermas raises against germline enhancements. Habermas argues that such enhancements would threaten the child's sense of a self-continuity between himself as he exists as a product of socialization, and himself as he existed in abstraction from this process, prenatally. Habermas claims that these enhancements would thus impinge on the child's autonomy, because ‘… the person can only see himself as the author of ascribable actions and the source, if he assumes self-continuity …’^26^ between himself as he exists in the world as a socialised being, and himself as he existed before entering the social world.

Accordingly, if a child's prenatal existence is affected by external social values, then that child will not be able to conceive of himself as having an identity beyond that which is determined by his socialization; the self of the individual who is prenatally enhanced would, Habermas claims, ‘slip away’^27^ Crucially, this would serve to undermine the enhanced individual's ability to regard himself as a member of an equal moral community, undermining, for Habermas the very ethics of the species.

### iii) The pre-emptive defence

It is crucial to acknowledge that in the course of making these arguments, Habermas appeals to two widely drawn distinctions in the enhancement debate, namely the distinction between therapy and enhancement, and the distinction between genetic and environmental enhancements. In doing so, he effectively provides a pre-emptive defence from some objections that liberals have offered against his arguments. However, the way in which Habermas draws these distinctions is often overlooked by his critics.

First, consider the distinction between therapy and enhancement. Supporters of enhancement often point out that we believe that it should be permissible to carry out genetic interventions that remove genes for specific genetic diseases. From this observation, some go on to claim that there is no important moral difference between this sort of therapeutic intervention, and an enhancing genetic intervention.^28^ However, Habermas explicitly rejects this view. He claims that therapeutic genetic modifications are permissible because we may presume consent on behalf of a modified individual to avoid a profound evil which is ‘unquestionably extreme, and likely to be rejected by all’^29^ but we cannot presume her consent to merely bring about some trait that the parent values; for Habermas, the latter is merely ‘a state that is desirable in terms of third party goals’^30^ and not a valid basis for presuming the consent of the enhanced subject.

Second, other philosophers have pointed out that we already enhance our children through altering their environment, and that there is no important difference between doing this and genetic enhancement.^31^ Once again though, Habermas explicitly rejects this view. Habermas argues that the crucial difference between the two is that modifying the child's environment involves a ‘communicative action’.^32^ As I explained in section I, Habermas claims that the self is partially formed by communication with others in a social environment. A crucial part of this is that as well as receiving communicated reasons we are also able to respond. In the case of enhancing an existing child's *environment*, Habermas suggests that parents include their child in a reciprocally responsive relationship in which the child can respond to their parent's choices. In contrast, germline enhancements offer no opportunity for a communicative process between the child and parent.^33^

Finally, it should also be acknowledged that, contrary to what some philosophers have claimed,^34^ Habermas' arguments do not rely on the fallacy of genetic determinism, as he himself explicitly points out.^35^

## III Towards A Liberal Response

The observations of the previous section should make it clear that since Habermas argues that there *is* a morally relevant distinction to be made between therapy and enhancement, as well as one between environmental and genetic enhancement, critics of Habermas cannot merely dismiss his arguments by simply stating that there are no morally relevant differences here; they must engage with his arguments to the contrary. I shall now sketch some ways in which liberals in the enhancement debate may adequately respond to Habermas' arguments. It should be stressed that the points I shall raise below are not intended to represent knock-down objections to Habermas' arguments, although I believe that they are powerful enough to at least raise considerable concerns about Habermas' objections. Rather, my primary aim here is to identify areas where liberal critics of Habermas' arguments can engage with supporters of these arguments in a manner that avoids the misunderstandings highlighted above.

### i) Responding to the argument from natality

There are two areas in which liberals might wish to question the argument from natality. The first concerns a possible source of tension between this argument and Habermas' position on the treatment/enhancement distinction, and the second concerns Habermas' metaphysics of the self.

As I explained above, for Habermas, natality represents a point of differentiation between the socialization fate of a person and their natural fate. In view of this, he argues that influencing a child prior to the point of natality will disrupt its sense of self-continuity, since it will be unable to conceive of itself as ever existing in abstraction from the forces of socialization. Supporters of enhancement might point out that Habermas' reliance on the concept of natality here seems in tension with his views on the treatment/enhancement distinction. Recall that Habermas claims that therapeutic modifications are morally permissible because we are warranted in presuming consent to remove a profound evil. However, the claim that therapeutic modifications are morally permissible seems to be in tension with his appeal to natality; surely carrying out therapeutic modifications threatens the concept that natality marks a point of differentiation of one's social and natural fates just as much as enhancing modifications; after all, in both cases, the contingency of the child's genetic make-up, which we have seen is necessary for the child's autonomy on Habermas' view, is taken away.

The point I am raising here is crucially different to the argument often raised by supporters of enhancement which points to the similarity of therapeutic and enhancing modifications in order to claim that one cannot consistently allow one whilst prohibiting the other. This is not what I am suggesting here; rather, I am drawing attention to the fact that Habermas' pre-emptive response to this argument, namely that there is a moral difference between a therapeutic genetic modification and an enhancing modification in terms of what we can validly presume consent for, seems to be in tension with his appeal to the concept of natality in his second argument against germline enhancements. If the contingency of one's genome is necessary for one's sense of self continuity, and *a fortiori* autonomy, then this seems to imply that a child who is the subject of a therapeutic genetic intervention, and whose genome is thereby not contingent, cannot have the sense of self-continuity that is necessary for autonomy.

Habermas could perhaps respond to this argument by conceding that his claim that *only* the conditions of nature like growth can ground autonomy is too strong. Instead, he should claim that a child who is subjected to therapeutic modifications can also conceive of herself in a manner that can ground autonomy, because her therapeutic modifications do not incorporate any sort of value which could be imposed on the ‘uncontaminated’ pre-natal self. However, supporters of enhancement might find this response problematic, if they believe that health is *itself* a value; moreover, they might also point out that health is a value that we shape in accordance with socializing influences of the sort that Habermas argues would threaten the future child's conception of herself as an autonomous agent.

There does seem to be some scope for this position. As the debate between the distinction between therapy and enhancement suggests, we do not have an objective, value free conception of what health is.^36^ Yet this is surely what is necessary if Habermas is to claim that one can carry out a therapeutic modification without thereby imposing certain values on the child, values that may be regarded as a way of exerting the forces of socialization on the pre-natal self. For instance, in modifying a child who would be born mentally retarded without intervention, we are implicitly endorsing the value of certain mental functions. More problematically, we might suppose that parents would choose to prenatally modify a child in order to ‘cure’ the child's deafness; yet, as has been suggested by recent cases, deaf parents would not necessarily regard the removal of the child's deafness here as ‘returning the child to health’, insofar as they do not regard deafness as an affliction.^37^ I cannot consider this complex case here; however, I raise it to show that if Habermas is to appeal to the concept of natality in his arguments against germline enhancements whilst endorsing some therapeutic modifications, supporters of enhancement might legitimately ask why the latter sort of modifications do not imply the imposition of the forces of socialization in the same way that enhancing modifications do. Even if Habermas is correct to claim that enhancing modifications imply certain expectations about the good life in a way that therapeutic modifications do not, this does not entail that therapeutic modifications are not value laden in *any* way.

A second point where supporters of enhancement might raise concerns about the argument from natality is Habermas' claim that a child's ability to conceive of themselves as a self-directed agent is undermined if their parents seek to enhance them pre-natally. One problem with this claim is it that we are *already* subject to environmental factors prior to our natality. For instance, our developmental well-being *in utero* depends on our mother's diet and overall health.^38^ Furthermore, studies have shown that *in utero* foetuses can be pre-natally stimulated by environmental factors (such as playing music) in a manner that can significantly benefit the child;^39^ this latter sort of benefit seems to be a clear example of a pre-natal, environmental *enhancement* (rather than therapy). However, we surely do not believe that these interventions are inimical to the child's ability to conceive of themselves as the author of their own life.

To conclude this discussion of the argument from natality, in arguing that we are only able to conceive of ourselves as being our ‘own person’ by virtue of the contingency of our genetic endowment, supporters of enhancement might reasonably argue that Habermas is doing us a disservice. Instead, they might claim that we are not merely the passive recipients of our socialization as Habermas implies when he talks about a socialization ‘fate’; we interpret and respond to the influences of our environment, and incorporate values upon critical reflection. Even though we might be passive recipients in our early developmental stages, there seems to be scope for the claim that we define ‘who we are’ and exercise our autonomy when we are able to critically evaluate our values and desires.

### ii) Responding to the empirical argument

There are two areas where supporters of enhancement might wish to raise concerns about Habermas' empirical argument. First, they might suggest that the parental expectations implicit in the child's enhanced traits are not really inescapable, and thus claim that it can be permissible to carry out enhancements if the child can reject them. Second, they might claim that we should just enhance those traits in which dissonant cases will not arise. I shall consider the latter, weaker reply first, before considering the stronger.

In *A Theory of Justice*, Rawls described what he termed ‘primary goods’, that is to say, goods which ‘… normally have a use whatever a person's rational plan of life’.^40^ Proponents of enhancement technologies have embraced this insight and claimed that if certain traits are useful for a wide range of life-plans, then enhancing these traits would not imply the parental expectation of a specific life-plan. Departing from Rawls, some proponents of this view term such traits ‘General Purpose Means’ (henceforth GPMs), defining them to be those traits which are:

… useful and valuable in carrying out nearly any plan of life or set of aims that humans typically have.^41^

The example of a GPM that Buchanan et al. provide is a good memory; we might also propose other attributes such as intelligence and the ability to socially interact as further putative GPMs. One way for liberals to respond to the empirical argument is to claim that we can legitimately presume consent for the enhancement of GPMs, since they do not incorporate the expectation of a specific life-plan, and are unlikely to lead to dissonant cases insofar as they are useful to any rational life-plan.^42^

Habermas (amongst others)^43^ rejects this view. As I highlighted in section II, Habermas claims that we are only warranted in presuming consent for therapeutic modifications insofar as they are ‘likely to be rejected by all’. As such, his reply to those who invoke the notion of GPM is that we cannot be sure that any putative GPM will always be of use to every individual; enhancing a GPM is deemed to be an unwarranted gamble. He asks:

Can parents wanting only the best for their child ever really presume to know all the circumstances … in which a brilliant memory … or high intelligence will prove a benefit for their child?^44^

He goes on to suggest cases in which a good memory can be a hindrance, since ‘… not being able to forget can be a curse’, and that ‘… sometimes an overloaded storage hinders us from dealing productively with new data to be taken in’.^45^

The second quote seems to misconstrue what a memory enhancement would involve; although having an overloaded storage might hinder our dealing productively with new data, the whole point of a memory enhancement would presumably be to free up space in our overloaded storage. However, let us take Habermas' general point; can liberals really be *sure* that enhancing GPMs will benefit the child by increasing their ability to practically realize any rational life plan that they might come to adopt?

Perhaps Habermas is right that we cannot be *absolutely* sure that enhancing a GPM will benefit the child; the child might come to adopt a life-plan that is so esoteric that GPMs will not be conducive to its practical realization. However, this in itself does not mean that an argument appealing to GPMs is a *non-sequitur* for liberals. After all, parents cannot predict with absolute certainty that *any* way in which they affect their child will increase the child's well-being. Rather, parents choose to affect their child in the ways that they do on the basis of what it is rational for them to believe will benefit their child. For instance, they believe that reading bed-time stories to a child is more likely to increase future literacy than it is to induce deep melancholia. However, they cannot be *certain* that this will be the case. Yet this does not represent an argument against reading bed-time stories. Parents are rationally justified in believing that reading their child stories will benefit them; the evidence in favour of this view is sufficient to justify the supposed ‘gamble’. Similarly, insofar as GPMs are claimed to normally have a use whatever a person's rational plan of life might be, it seems most rational for parents to believe that enhancing a GPM would benefit their child by increasing their ability to act effectively in pursuit of their ends.

Furthermore, liberals might point out that refusing to countenance the possibility of GPMs is to ignore the evidence that we have of GPMs being conducive to well-being which we can garner by considering existing people who excel in GPM traits. Perhaps opponents of enhancements neglect to acknowledge the obvious potential benefits of increasing GPMs because they feel that the onus is on them to highlight the less obvious point that GPMs might be detrimental in some cases. This might be right; however, this should not blind us to the fact that there is a good case for believing that enhancing certain GPMs *would* benefit the child, given the utility of these traits to a wide range of rational life plans.

The above reply is weak in the sense that it does not rule out the possibility of dissonant cases. As such, if we believe that the self-alienation that agents in dissonant cases suffer is as bad as Habermas claims, then it might be argued that we should not risk enhancing GPMs on the basis of expected utility; even if such enhancements have a high chance of benefiting the child, it might still be argued that we should not enhance GPMs because the negative value of the dissonant cases which they have a (very) small chance of leading to is so very high. As such, although an appeal to GPMs in response to Habermas is not a *non-sequitur*, neither is it a knock-down objection to Habermas' empirical argument. However, the issue of GPMS is one, I suggest, upon which supporters of Habermas arguments and liberals in the enhancement debate can engage in a meaningful debate.

I shall turn now to a stronger reply that liberals might legitimately make to the empirical argument, which claims that the parental expectations implicit in a child's enhanced traits are not irrefutable and inescapable in the way that Habermas claims. Recall that, for Habermas, these parental expectations are irrefutable and inescapable for two reasons. First, they do not involve the child in a ‘communicative relationship’ with the parent, and second, they are inescapable insofar as the child incarnates those expectations. I shall consider each reason in turn.

It seems that there is scope for questioning whether Habermas' views on the communicative relationship between parent and child form an adequate basis for his claim that a child would regard her enhanced traits as irrefutable and inescapable. In the first place, it is not clear that all environmental modifications involve a communicative relationship between the parent and child.^46^ Of course, Habermas would likely respond to this observation by pointing out that post-natally enhanced children at least normally have the *opportunity* to express their disapproval of their parents' dictates (even if they are ignored). However, liberals might yet argue that genetic modifications need not preclude a communicative relationship between a parent and child in which the child can express her disapproval. As Bostrom points out, Habermas' assumption that germline modifications would be an unanswerable fact is perhaps unwarranted, since:

… our descendents … will presumably be far more technologically advanced than we are … (and would) … not lack the means to … frustrate our designs.^47^

However, even if liberals make this sort of response, Habermas has still highlighted some areas of legitimate concern. First, if such means of reversing enhancements remained unavailable, then the possibility of dissonant cases would remain; and Habermas is surely right to argue that these cases are deeply problematic. We can perhaps begin to imagine the distress that such dissonant cases would involve by considering the seemingly comparable cases of those who suffer from gender dysphoria, and certain circumcised men who report feeling alienated from their bodies.^48^

Furthermore, even if the enhanced child could reverse her enhancement, presumably she would only be able to do so when her consent is deemed valid, after a number of years of living in her private embodied prison. It might be replied that the child's parents would surely consent on their behalf, and this might be so in some cases. However, there will still be a period of considerable distress for the child before their parents find out. The child may even feel unable to tell their parents of their discomfort; the risk of this is likely to be augmented if the child's parents have spent a considerable sum of money in enhancing them. Indeed, some parents may just not be willing to consent to their child's reversing their enhancement.

I do not wish to fall into the trap of making speculative empirical claims; however, the points raised here *are* potential risks, and we have reason to avoid these outcomes. Nevertheless, the above reflections suggest that the mere possibility of dissonant cases may not be sufficient to render all germline enhancements morally impermissible.

## IV Conclusion

In this article, I have joined others in claiming that Habermas' arguments against germline enhancement have not received adequate critical attention from many prominent liberal bioethicists. However, I have also suggested areas in which liberal bioethicists can raise legitimate concerns about two of Habermas' arguments. To reiterate, the points I have made here are not intended to be knock-down objections against these arguments. Rather, I hope to have highlighted areas in which supporters of Habermas and liberal bioethicists can engage in a meaningful debate on this most salient of issues.

## Footnotes

1 A. Buchanan. *Beyond Humanity*. Oxford: Oxford University Press; 2011. p. 6.

2 J. Harris. *Enhancing Evolution*. Princeton: Princeton University Press; 2007. p. 141.

3 B.G. Prusak. Rethinking ‘Liberal Eugenics’: Reflections and Questions on Habermas on Bioethics. *Hastings Cent Rep* 2005; 35(6): 32.

4 J. Habermas. *Between Naturalism and Religion*. Polity Press; 2008. p. 160.

5 J. Habermas. *The Future of Human Nature*. Polity Press; 2003. p. 50.

6 Ibid: 57.

7 Ibid: 14.

8 Habermas, *op. cit.* note 4, p. 170.

9 Habermas, *op. cit.* note 5, p. 34.

10 Habermas, *op. cit.* note 4, p. 161.

11 Habermas, *op. cit.* note 5, p. 6.

12 Habermas, *op. cit.* note 5, p. 59.

13 Herissone-Kelly argues that Habermas' arguments can be extended to somatic interventions. See P. Herissone-Kelly. Habermas, Human Agency, and Human Genetic Enhancement; The Grown, The Made, and Responsibility for Actions. *Cam Q Healthc Ethics* 2012: 21.

14 The distinction I draw between the empirical argument and the argument from natality maps on to the distinction that Häyry draws between two vocabularies that he claims Habermas employs, namely the ‘lexicon of freedom’, and the glossary of dignity. M. Häyry. Protecting Humanity; Habermas and His Critics on the Ethics of Emerging Biotechnologies. *Cam Q Healthc Ethics* 2012; 21: 215.

15 E. Malmqvist. Reprogenetics and the ‘Parents Have Always Done It’ Argument. *Hastings Cent Rep* 2011; 41: 1.

16 N. Bostrom. In Defence of Posthuman Dignity. *Bioethics* 2005; 19(3): 212.

17 See Malmqvist, *op. cit.* note 15.

18 See G. Dworkin. *The Theory and Practice of Autonomy*. Cambridge: Cambridge University Press; 1988. ch. 5.

19 Habermas, *op. cit.* note 5, p. 51.

20 See J. Glover. *Choosing Children: Genes, Disability and Design*. Oxford: Oxford University Press; 2006. p. 72.

21 Habermas, *op. cit* note 5, p. 61.

22 Ibid: 63.

23 Ibid: 42.

24 Prusak, *op. cit.* note 3, p. 35.

25 Ibid: 59.

26 Ibid: 59.

27 Ibid: 60.

28 For example, see Harris, *op. cit.* note 2, p. 139 and Agar. Liberal Eugenics. *Public Aff Q* 1998; 12(2): 172–173.

29 Habermas, *op. cit.* note 5, p. 43.

30 Ibid, p. 52.

31 N. Agar. *Liberal Eugenics*. Wiley Blackwell; 2004. ch 6.

32 Habermas, *op. cit.* note 5 p. 51.

33 Ibid: 51–53.

34 For example, see I. De Melo-Martín. Designing People: A Post-Human Future? In: P.E. Vermaas, P. Kroes, A. Light & S.A. Moore, editors. *Philosophy and Design*. Dordrecht: Springer; 2008. pp. 198–199; and D.T. Wasserman. My Fair Baby: What's Wrong with Parents Genetically Enhancing Their Children? In: V.V. Gehring, editor. *Genetic Prospects: Essays on Biotechnology, Ethics, and Public Policy*. Lanham: Rowman & Littlefield; 2003. p. 106.

35 Habermas, *op. cit.* note 5, p. 53.

36 J. Savulescu, A. Sandberg & G. Kahane. Well-Being and Enhancement. In: J. Savulescu, G. Kahane, R. ter Meulen, editors. *Enhancing Human Capabilities*. Chichester: Wiley Blackwell; 2011.

37 J. Savulescu. Deaf lesbians, ‘designer disability’, and the future of medicine. *BMJ* 2002: 325.

38 See L. Poston, L. Harthoorn, E. van der Beek & M. Hanson. Obesity in Pregnancy: Implications for the Mother and Lifelong Health of the Child. A Consensus Statement. *Pediatr Res* 2011: 69.

39 See C. Panthuraamphorn. Prenatal infant stimulation program. In: T. Blum, editor. *Prenatal Perception, Learning and Bonding*. Berlin & Hong Kong: Leonardo; 1993.

40 J. Rawls. *A Theory of Justice (Revised Edition)*. Belknap Press; 2000. p. 54.

41 A. Buchanan, D. Brock, N. Daniels & D. Wikler. *Chance Versus Choice*. Cambridge: Cambridge University Press; 2000. p. 167.

42 D. Fox. The Illiberality of Liberal Eugenics. *Ratio* 2007: 20(1).

43 See S. Varga. Habermas' ‘Species Ethics’, and the Limits of ‘Formal Anthropology’. *Crit Horizons* 2011; 12(1): 286.

44 Habermas, *op. cit.* note 5, p. 85.

45 Ibid: 85.

46 See Agar, *op. cit.* note 31, p. 117 and Wasserman, *op. cit.* note 34, p. 105.

47 Bostrom, *op. cit.* note 16, p. 211.

48 See T. Hammond. A preliminary poll of men circumcised in infancy or childhood. *BJU International* 1999; 83, Suppl. 1.

